# Genotype x environment interaction and genetic gain for grain yield and grain quality traits in Turkish spring wheat released between 1964 and 2010

**DOI:** 10.1371/journal.pone.0219432

**Published:** 2019-07-18

**Authors:** Ajit Nehe, Beyhan Akin, Turgay Sanal, Asuman Kaplan Evlice, Rıza Ünsal, Nazım Dinçer, Lütfü Demir, Hatice Geren, Ismail Sevim, Şinasi Orhan, Sadiye Yaktubay, Ali Ezici, Carlos Guzman, Alexey Morgounov

**Affiliations:** 1 International Maize and Wheat Improvement Center (CIMMYT), Emek, Ankara, Turkey; 2 Central Research Institute for Field Crops, Yenimahalle, Ankara, Turkey; 3 Aegean Agric. Research Institute, Menemen, Izmir, Turkey; 4 East Mediterranean Agric. Research Institute, Dogankent, Yüregir, Adana, Turkey; 5 Maize Research Station, Hanlı, Arifiye, Sakarya, Turkey; 6 International Maize and Wheat Improvement Center (CIMMYT), México City, Mexico; Institute of Genetics and Developmental Biology Chinese Academy of Sciences, CHINA

## Abstract

The study was conducted to determine the effects of genotype (G), environment (E), their interaction (GEI) and genetic gain on yield and grain quality traits in Turkish spring wheat cultivars released between 1964 and 2010. We conducted a multi-environment trial at three testing locations: Adana, Adapazarı, and Izmir, during the 2009, 2011 and 2013 cropping seasons and tested 35 cultivars released by the respective breeding programs. Allelic variations of high and low molecular weight glutenin subunits (HMW-GS and LMW-GS) and 1B/1R translocation was also determined and evaluated in all cultivars. Comparing yield across three locations, Adana (6416 kg ha^-1^) yield was relatively higher than in Izmir (5887 kg ha^-1^) and Adapazarı (5205 kg ha^-1^) (P<0.001). Overall, GY was influenced by the varieties, testing location and breeding programs (P<0.001). Cultivars from Izmir breeding program performed relatively better (6174 kg ha^-1^) than those from Adana (5996 kg ha^-1^) and Adapazarı (5351 kg ha^-1^) (<0.001). We recommend Ziyabey-98, Menemen, and Basribey-95, for stable grain yield in spring wheat production across the studied regions because of their wide adaptability, and Pamukova-97 for future breeding to improve grain quality parameters. We found three breeding programs have successfully increased the grain yield and quality traits for 46 years. As a group, cultivars released after 2000 had the highest yield indicating breeding progress. Genetic gain for GY was 30.9 kg ha^−1^ per year from 1964 with annual increase compared to the yield of older cultivar Akova B-2 (4102 kg ha^-1^) which constitutes a 0.75% rate of genetic gain. Improvement in grain quality was related to change in protein composition rather than an increase in protein content whereas yield improvement seems to mainly be related to increases in test weight and 1000 kernel weight. High molecular weight glutenin subunit (HMW-GS) 5+10 showed an increase in frequency whereas 2+12 showed a decrease over the breeding period.

## 1. Introduction

Wheat is a very important crop for the Turkish economy. As one of the biggest wheat flour exporters in the world, Turkey exported 3.5 million tons of wheat flour in 2016 [1]. However, in the last few years Turkish wheat production has shown instability. Overall in 2017, Turkish wheat production was 21 million tons with average yield of 2.8 kg ha^-1^ which is comparatively low to European average 4.4 kg ha^-1^ [2]. In the last 15 years, Turkish dependability on quality wheat import to fulfill the domestic industrial demand is increased by almost 4 times. To ensure reliable and stable production of high-quality wheat, the development of wheat cultivars (G) with a consistent high yield in different environments (E) along with good grain quality is necessary. There is a high demand for cultivars with hard endosperm and strong and extensible gluten, especially in the mechanized bread making industry. Developing such cultivars is a challenging and an important objective for wheat breeding programs in Turkey. Knowledge of the genetic diversity of traits and their interaction with the environment is a prerequisite for any such breeding program. Understanding the wheat grain at both the molecular and field level is critical for identifying grain quality traits, as these traits depend on the genetics of the cultivar and are influenced by environment.

The physical characteristics of grain, such as test weight (TW) and thousand kernel weight (TKW), are important as they are indicators of potential processing quality. TW—the weight of a specific volume of grain—is an indicator of the bulk density of the grain and helps in wheat grading and trade. Some cultivars have higher TW than others under similar growing conditions because of G x E interaction [3]. TKW—the weight of a thousand healthy, whole wheat grain, indicates kernel size and density, and is highly influenced by genotype, location and agricultural practice [4]. Overall, depending on environmental conditions, TKW could be a more reliable indicator for expected flour yield than test weight to industrial millers, as a strong correlation has been found between TKW and flour yield [5]. In some conditions there is a high corrections between TW and total extractable flour [6].

For grain composition, protein content (PC) is a fundamental parameter of wheat and an indicator of the end-use quality of wheat products. PC is more affected by environment than by genotype [7,8]. Grain PC and protein quality determine wheat flour quality and, along with stable flour composition, are desirable traits in the wheat flour industry [7]. Protein quality is mainly determined by the composition of gluten molecules. Gluten, which is formed by the storage proteins of wheat grain and located in the endosperm, is the key determinant for the functional characters of dough. These functional properties are usually evaluated with mixograph, alveograph and farinograph analysis [9,10]. Dough made from a wheat genotype with strong gluten usually exhibits more resistance to stretching and breakdown due to overmixing than dough with weak gluten. The mixing curve obtained from a mixograph analysis shows the ideal time of development, tolerance to overmixing, and other dough characteristics which are used to estimate the bake absorption [11].

At the molecular level, most important components of the gluten are: gliadin and glutenin, which are determinant for most of quality traits in the process of bread making [12]. Gliadin and glutenin are found in a 2:3 ratio in wheat protein [13,14]. The gliadin fraction confers mainly the extensibility and viscosity of the gluten, while glutenin affects its elasticity. Glutenin is composed of low molecular weight glutenin subunits (LMWGSs) and high molecular weight glutenin subunits (HMWGSs) contributes almost 30% to 40% of the total protein in the flour. In previous studies it has been found that HMWGSs have more effect on overall quality of bread as compared to LMWGSs, even though their relative proportion is only 10% of the total storage proteins. LMWGSs constitute almost 40% in overall composition [15]. The HMWGSs are regulated by allelic genes (*Glu-A1*, *Glu-B1*, and *Glu-D1*) located on the long arms of chromosomes 1A, 1B and 1D respectively [16]. LMWGS are regulated by three loci, *Glu-A3*, *Glu-B3*, and *Glu-D3* [17].

Wheat quality is also influenced by the presence or absence of wheat-rye 1B/1R chromosome translocation. Worldwide, breeders use wheat-rye 1B/1R chromosome translocation because it contains disease resistance genes and contributes to yield. Unfortunately, wheat cultivars with 1B/1R translocation were reported to have poor quality of bread associated with dough stickiness and poor mixing tolerance [18]. There is a need, therefore, to study and improve the genetic diversity of the gluten proteins in Turkish bread wheat cultivars [19].

The quality traits interact with the environment, so it is important to evaluate and quantify to what degree factors like the environment (E) and genotype x environment interaction (GEI) are responsible for phenotypic variation in the traits. The effect of E on certain quality parameters varies, but it is generally stronger on protein content (PC) and protein related parameters [20]. From the breeding point of view, it is important to understand the masking effect of environment relative to actual heritable traits by determining the GEI and identify traits which shows stable effect across different environment [21]. Moreover, it is important to see whether the breeding programs are successful by evaluating their genetic gains. Such evaluation can help in identifying the traits that contribute to yield improvement and developing the future breeding strategies. [22]. For this purpose, breeding programs in many countries select historical set of cultivars and study them in different environment to determine the genetic gains achieved through breeding and selection over particular breeding period [23–25]. Wheat genetic gains in Turkey have been recently summarized for irrigated winter wheat [26], rainfed winter wheat [27] and spring wheat [28]. The spring wheat research [28] focused on genetic gains for yield and its components. The analysis presented in this paper used the same experimental data with a focus on GEI and genetic gains for grain quality parameters.

The objectives of this research were: i) to study the effect of G, E and GEI towards the variation in GY and bread making quality traits across three location amongst 35 Turkish spring wheat cultivars, ii) understand the association between GY and quality traits along with agronomic traits, iii) to determine the effect of glutenin subunits (HMW-GSs and LMW-GSs) contribution towards the grain quality iv) to identify the best wheat cultivars suitable for a particular location or region, based on GY and quality traits.

## 2. Materials and methods

### 2.1 Experiment design

The study was conducted at East-Medditerranean Agricultural Research Institute (Adana) Maize Research Institute (Adapazarı), and Aegean Agricultural Research Institute (Izmir), during three seasons: 2009, 2011 and 2012. Thirty-five spring bread wheat cultivars released from 1964 to 2010 by the breeding programs at three institutes were selected (Table 1). The criteria for cultivar selection were to include cultivars commercially important to Turkish economy and have contribution to overall wheat production. The experiment was conducted in a randomized block design, with three replications on all the sites × years. Plots were six rows of 7.0 m × 1.2 m. The distance between rows was 20 cm and 450 seeds were sown per square meter. Fertilizers applied were 100 kg of phosphorus (P) and 39 kg of nitrogen (N) per hectare at the time of planting, and an additional 50 kg ha^−1^ at tilling. In all locations, irrigation was given with 50 mm of amount of water once or twice during growing season. For weed control, herbicide 2,4-D at the rate of 1.5 L ha^−1^ was applied before stem elongation. Crop was harvested at maturity with a combine plot harvester, leaving a 1.0 m edges at the beginning and ends of plots (i.e. a harvested plot size of 5.0 m × 1.2 m).

**Table 1:** Characteristics of spring wheat cultivars released in Turkey between 1964 and 2010 and tested at three sites in 2009, 2011 and 2012 in Turkey.

No	Cultivars	Pedigree	Release year	Origin
Cultivars released by East Mediterranean Agricultural Research Institute, Adana				
27	Orso	FUNO/PRODUTTORE	1977	Italy
30	Pandas	Orso//BEZ/S1//GEN7/Marzotto	1985	Italy
11	Çukurova 86	BLUEBIRD/KALYANSONA	1986	CIMMYT
34	Yüreğir 89	HD-1220/3*KALYANSONA//NACOZARI-76	1989	CIMMYT
13	Doğankent 1	FLK/HORK	1991	CIMMYT
32	Seyhan 95	JUP/BJY//URES (KAUZ'S' = BACANORA)	1995	CIMMYT
1	Adana 99		1999	
10	Ceyhan 99	BJY/COC	1999	CIMMYT
20	Karatopak	TSI/VEE//SERI-82	2006	Turkey
28	Osmaniyem	TUJ/ONELTO	2006	CIMMYT
2	Aday 1	CHEN/AEGILOPS SQUARROSA(TAUS)//BCN/3/2*KAUZ	2010*	CIMMYT
Cultivars released by Maize Research Station, Adapazarı				
3	Akova B-2		1964	Turkey
4	Aköz-867	RIETI/WILHELMINA//AKAKOMUGI	1968	Turkey
31	Sakarya-75	CIANO-67/PENJAMO-62//CIANO-67/SIETE-CERROS-66	1967	CIMMYT
22	Libellula	TEVERE/GIULIARI (1482-54-3)//SAN-PASTORE	1983	Italy
16	İrnerio	PRODUTTORE/MANITOBA	1985	Italy
29	Pamukova-97	VEERY/PAJONAL	1997	CIMMYT
19	Karacabey-97	VEERY #5/PAVON F 76/3/GOLDEN VALLEY/AZTECA 67//MUSALA	1997	CIMMYT
7	Bandırma-97	BOW/PRL	1997	CIMMYT
26	Momtchill	NS11-36/AU	2000	Bulgaria
33	Tahirova-2000	Prl/Vee#6//Myna/Vul	2000	CIMMYT
15	Hanlı	OK82282//BOW/NKT/3/F4105	2007	TCI**
9	Beşköprü	362K2.111/6/NKT/5/TOB/CNO67//TOB/8156/3/CAL//BB/CNO67/4/TRM	2007	CIMMYT
Cultivars released by Aegean Agricultural Research Institute, Izmir				
12	Cumhuriyet 75	ISWRN-297//SONORA 64/ANDES 64 A/3/FEDERATION*2/AUS-11338	1975	CIMMYT
6	ATA81	KVZ/CUT75	1981	Turkey
17	İzmir81	Not available	1985	CIMMYT
23	Maramara86	AU//KAL/BB/3/WOP	1986	CIMMYT
18	Kaklic88	KVZ/BUHO//KAL/BB	1988	CIMMYT
21	Kasifbey95	HORK/YAMHILL//KALYANSONA/BLUEBIRD	1995	CIMMYT
8	Basribey95	JUPATECO F 73/BLUEJAY//URES T 81	1995	CIMMYT
14	Gonen98	8156 RESELECTION/MARA//BLUEBIRD	1998	Turkey
35	Ziyabey98	ND/VG9144//KAL/BB/3/YACO/4/VEE 5	1998	CIMMYT
25	Meta2002	NORDDEPREZ/VG9144//KALYANSONA/BLUEBIRD/3/YACO/4/VEERY #5	2002	CIMMYT
24	Menemen	SUPER KAUZ	2004	CIMMYT
5	Alibey	KAUZ*2//SAP/MON/3/KAUZ	2004	CIMMYT

* Line Aday 1 released as variety in 2010 but included in this experiment in 2009.

**TCI–Turkey-CIMMYT-ICARDA International Winter Wheat Improvement program.

The data was recorded in the field and post-harvest for the following traits: grain yield (GY), thousand kernel weight (TKW), test weight (TW), wet gluten content (WGC), protein content (PC), Zeleny sedimentation value (ZS), modified Zeleny sedimentation value (MZS), alveograph energy value (Alv.W), mixograph developmental time (MDT), mixograph mixing tolerance (MMT) and mixograph softening degree (MSD). We analyzed quality traits only on the first replication at all sites and years and evaluated yield components using 10 random plants from each plot and replication. TKW was calculated by the weight of 20 g and counting the kernels. As PC and TW are highly correlated with GY, the values were adjusted with GY. Similarly, Zeleny sedimentation value and wet gluten values were adjusted with the adjusted PC values, by multiplying trait values by slope values calculated by regression analysis between two traits and adding values of Y intercept.

### 2.2 Quality analysis

Grain samples were cleaned using dockage (Quator, Trippette & Renaud, France) and the physical characteristics of the grain were determined—such as TW and TKW [29]. After the physical analysis, we ground 50 g of each subsample in a grinder (Perten 3100, Huddinge, Sweden) with a 1.0 mm screen. Moisture content of the samples was determined by oven drying at 130°C for 2 hours, according to of the American Association Cereal Chemists (AACCI) approved Method 44-15A [30]. Nitrogen content was determined using the dumas method (Velp-NDA 701 Usmate, Italy) and multiplied by nitrogen to protein conversion factor 5.7 (N x 5.7), according to the AACCI Method 46–30 [30]. After overnight grain sample drying up to 15% moisture content, grains were milled using a Buhler Pneumatic Laboratory Mill (MLU-202 Uzwil, Switzerland) according to the official methods of the AACCI [30]. Zeleny sedimentation value was determined according to the International Association for Cereal Science and Technology (ICC) Method 116/1 [31] and a modified Zeleny sedimentation test (2 hours lag time between mixing and scoring to evaluate the effect of Sunny pest damage to grain) was also used [32]. Wet gluten content was obtained by using Glutomatic system (Perten Instument, Huddinge, Sweden) according to AACCI Method 38-12A. The Alveograph energy value (W) of the flour was determined by using an alveograph (Chopin, Villeneuve, France) instrument according to AACCI Method 54-30A. Mixograph parameters of the flour samples were determined by using a mixograph device (National Manufacturing, Lincoln. USA) with 35g of flour with added water according to AACCI Method 54-40A. The mixograph results were evaluated for mixograph development time (MDT), mixing tolerance (MMT), and softening degree (MSD) [33, 34, 3].

HMW-GSs and LMW-GSs were separated by sodium dodecyl-sulfate polyacrylamide gel electrophoresis (SDS-PAGE) based on the extraction method [35], with some modifications. We determined the presence of the 1B/1R translocation with SDS-PAGE of alcohol-soluble and alcohol insoluble protein extracts, detecting the presence of Sec-1 secalins in the first test and then the presence of the Glu-B3j allele in the second [36].

### 2.3 Climate and weather conditions at experimental sites

The Adana, Adapazarı and Izmir provinces in which the trials were conducted are located in mild coastal areas with high rainfall (>500 mm), where spring wheat is planted in autumn and is normally rotated with maize, cotton or vegetables. The Adana region is characterized by higher rainfall and higher air temperatures, resulting in a shorter season and higher yields than Izmir or Adapazarı. During this experiment, Adana experienced greater variation in rainfall compared to other locations. Average seasonal air temperatures from November to May were 14.3°C for Adana, 11.5°C for Adapazarı and 12.5°C for Izmir.

### 2.4 Statistical analysis

Analysis of variance (ANOVA) across the years was produced with the help of GenStat version 19 (www.genstat.com, VSN International Ltd, Hemel Hempsted, UK) according to a randomized block design with replications for yield components and without replications for quality traits (Table 2). We conducted additional ANOVA to see the effect of year, breeding program and testing location and their interactions also using GenStat (S1 Table). We carried out regression analysis to determine the genetic progress over time, with years as the independent variable (x) and productivity or quality traits as dependent variables (y), applying the standard linear regression analysis to 3-year cultivar means for the 35 spring bread wheat cultivars. The regression coefficients are presented for the variables when necessity. We used regression coefficient (b) to determine the genetic gain, and the least significant difference (LSD) method to determine the significance. We carried out GGE biplot procedures to test associations between traits using the R software Version 3.0–2 (http://www.R-project.org/), and used the GGEbiplotGUI package tool to identify the “ideal genotype” and rank the cultivars. [37].

**Table 2:** Variance and significance effect of cultivars, location and their interaction to grain yield and quality parameters for 35 Turkish spring wheat cultivars studied in 2009, 2011 and 2012.

Source of variation	df	Grain yield	1000 Kernel weight	Test weight	Protein content	Wet gluten content	Zeleni sedimentation	Alveograph W value	Mixograph development time	Mixograph mixing tolerance	Mixograph
Variety	34	22.0 ***	32.5 ***	22.5 ***	1.55 NS	19.6 ***	19.1 ***	49.1 ***	22.5 **	37.0 ***	19.9 ***
Location	2	16.7 ***	23.2 ***	3.32 ***	77.1 ***	25.7 ***	24.8 ***	21.3 ***	3.32 **	8.52 ***	9.90 ***
Location x Variety	68	10.8 NS	2.84 NS	4.85 ***	5.66 NS	6.66 NS	7.92 NS	14.8 NS	4.84 NS	7.09 NS	13.6 NS
Residual	210	50.6	41.5	70.1	15.7	48.1	48.2	43.2	70.1	48.3	57.4

NS = Non-significant

*** = <0.001

** = <0.05

* = <0.5 level of significance. Degree of freedom (df), Cultivars (Vari), testing location (Loc), grain yield (GY), thousand kernel weight (TKW), test weight (TW), protein content (PC), wet gluten content (WGC), Zeleny sedimentation test value (ZS), alveograph energy value-W (Alv.W), mixograph developmental time (MDT), mixograph mixing tolerance (MMT), mixograph softening degree (MSD).

## 3. Results

### 3.1 ANOVA

ANOVA results for cultivars, environment and their interaction are shown in Table 2. Combined ANOVA results for year, breeding program and testing location and their interaction are presented in S1 Table. An overall analysis of variance showed that there was significant difference between cultivars and between testing locations for all the traits except for PC for cultivars. Two-way interaction of cultivars x location was significant only for TW.

### 3.2 Grain yield, yield components and quality traits

There was wide and significant variation among the cultivars for yield and quality parameters (Table 3 and S2 Table). Average yield across all cultivars and years ranged from 4102 to 6657 kg ha^-1^ with an average of 5836 kg ha^-1^. For GY there was a significant difference between cultivars (P<0.001) and locations (P<0.001) but not a significant difference for interaction. TKW varied between 35.9–47.1 g with an average of 40.7 g across the cultivars, years and locations. TW varied from 73.5 to 75.4 kg hL^-1^ (mean = 74.7 kg hL^-1^) and was strongly influenced by the environment, with location contributing 97.6% of total variance (S1 Table). The yield at the Adana site (6416 kg ha^-1^) was relatively higher than at Izmir (5887 kg ha^-1^) and Adapazarı (5205 kg ha^-1^) (P<0.001).

**Table 3:** Average values across year, testing location and breeding program for grain yield and quality parameters of 35 spring wheat cultivars developed by three breeding programs (Adana, Adapazarı and Izmir) between 1964 and 2010.

Traits	Data years	Breeding center	Testing location			Mean BP	LSD (BP/TL)
			Adana	Adapazarı	Izmir		
Grain yield (kg ha^-1^)	2009, 2011, 2012	Adana	6335	5481	6173	**5996**	214 ***
		Adapazari	6330	4487	5236	**5351**	
		Izmir	6577	5670	6276	**6174**	
		**Mean TL**	**6416**	**5205**	**5887**	**5840**	**288** ***
1000 kernel weight (g)	2011, 2012	Adana	42.8	37.3	44.2	**41.4**	0.94***
		Adapazari	43.3	38.1	43.5	**41.6**	
		Izmir	40.7	35.5	49.5	**41.9**	
		**Mean TL**	**42.2**	**37.1**	**42.7**	**41.6**	**1.14** ***
Test weight (kg hL^-1^)	2009, 2011, 2012	Adana	78.6	66.8	79.2	**74.9**	0.16 ***
		Adapazari	78.6	66.6	77.9	**74.4**	
		Izmir	78.7	66.8	79.4	**74.9**	
		**Mean TL**	**78.6**	**66.7**	**78.8**	**74.7**	**0.20** ***
Protein content (%)	2009, 2011, 2012	Adana	12.5	14.4	13.9	**12.5**	
		Adapazari	12.5	14.1	14.0	**14.3**	
		Izmir	12.3	14.5	13.9	**13.9**	
		**Mean TL**	**12.4**	**14.3**	**13.9**	**13.6**	**0.12** ***
Wet gluten content (%)	2009, 2011	Adana	26.7	28.4	30.8	**28.6**	0.76 ***
		Adapazari	26.3	29.2	32.6	**29.4**	
		Izmir	24.12	27.5	30.0	**27.2**	
		**Mean TL**	**25.7**	**28.4**	**31.2**	**28.4**	**1.02** ***
Zeleny sedimentation (ml)	2009, 2011, 2012	Adana	34.0	38.8	36.7	**36.5**	0.86 ***
		Adapazari	33.5	39.5	39.2	**37.4**	
		Izmir	31.2	38.1	35.5	**34.9**	
		**Mean TL**	**32.9**	**38.8**	**37.1**	**36.3**	**1.16** ***
Alveograph.W values (10^-4^joule)	2009, 2011	Adana	189	222	174	**195**	14.9 ***
		Adapazari	187	229	176	**197**	
		Izmir	160	201	151	**170**	
		**Mean TL**	**178**	**218**	**167**	**188**	**14.7** ***
Mixograph development time (min)	2009, 2011, 2012	Adana	5.39	3.91	4.27	**4.52**	0.51 ***
		Adapazari	5.53	4.72	5.14	**5.13**	
		Izmir	5.36	4.38	4.63	**4.79**	
		**Mean TL**	**5.93**	**4.76**	**5.14**	**4.81**	**0.77**
Mixograph mixing tolerance (°)	2009, 2011, 2012	Adana	115	114	111	**114**	2.37 ***
		Adapazari	149	148	140	**146**	
		Izmir	148	148	141	**145**	
		**Mean TL**	**150**	**149**	**143**	**134.9**	**2.61** ***
Mixograph softening degree (MU)	2009, 2011, 2012	Adana	7.1	6.9	8.9	**7.6**	1.04 ***
		Adapazari	10.4	9.8	13.8	**11.3**	
		Izmir	7.9	11.6	12.4	**10.6**	
		**Mean TL**	**9.16**	**10.2**	**12.7**	**9.9**	**1.21** ***

*** = <0.001; ** = <0.05; * = <0.5 level of significance. Adana (ADANA), Adapazarı (ADPZ), Izmir (IZMIR), testing location (TL), breeding program (BP).

Overall grain yield was also influenced by the breeding program (P<0.001). Across all environments, cultivars from Izmir breeding program (6174 kg ha^-1^) performed relatively better than those developed in Adana (5996 kg ha^-1^) and Adapazarı (5351 kg ha^-1^) (<0.001). The physical grain properties like TKW and TW, which are important indicators of flour yield, were higher in cultivars bred in Izmir than in cultivars bred in two other locations (P<0.001). Cultivars from Izmir breeding program showed relatively higher TKW and TW than other cultivars (Table 3).

The average values of protein content, Zeleny sedimentation value, wet gluten content and alveograph energy value were 13.5%, 36.3 ml, 47.1 ml, 27.2% and 188x10^-4^ joule respectively. Mixograph development time and mixing tolerance ranged from 3.28 minutes to 8.61 minutes and 135°C to 158°C, respectively. Overall, the cultivars at Adapazarı location performed best for protein content, Zeleny sedimentation value, alveograph energy value-W and. For the germplasm performance, top three cultivars with higher alveograph energy values were Pamukova-97, Karacabey-97 and Bandirma-97. For wet gluten content—Osmaniyem, Pandas and Akova B-2 and for mixograph development time—Pamukova-95, Adana-99, Ceyhan-99 and Çukurova-86.

### 3.3 Genotype and genotype x environment biplot analysis

**Fig 1:**
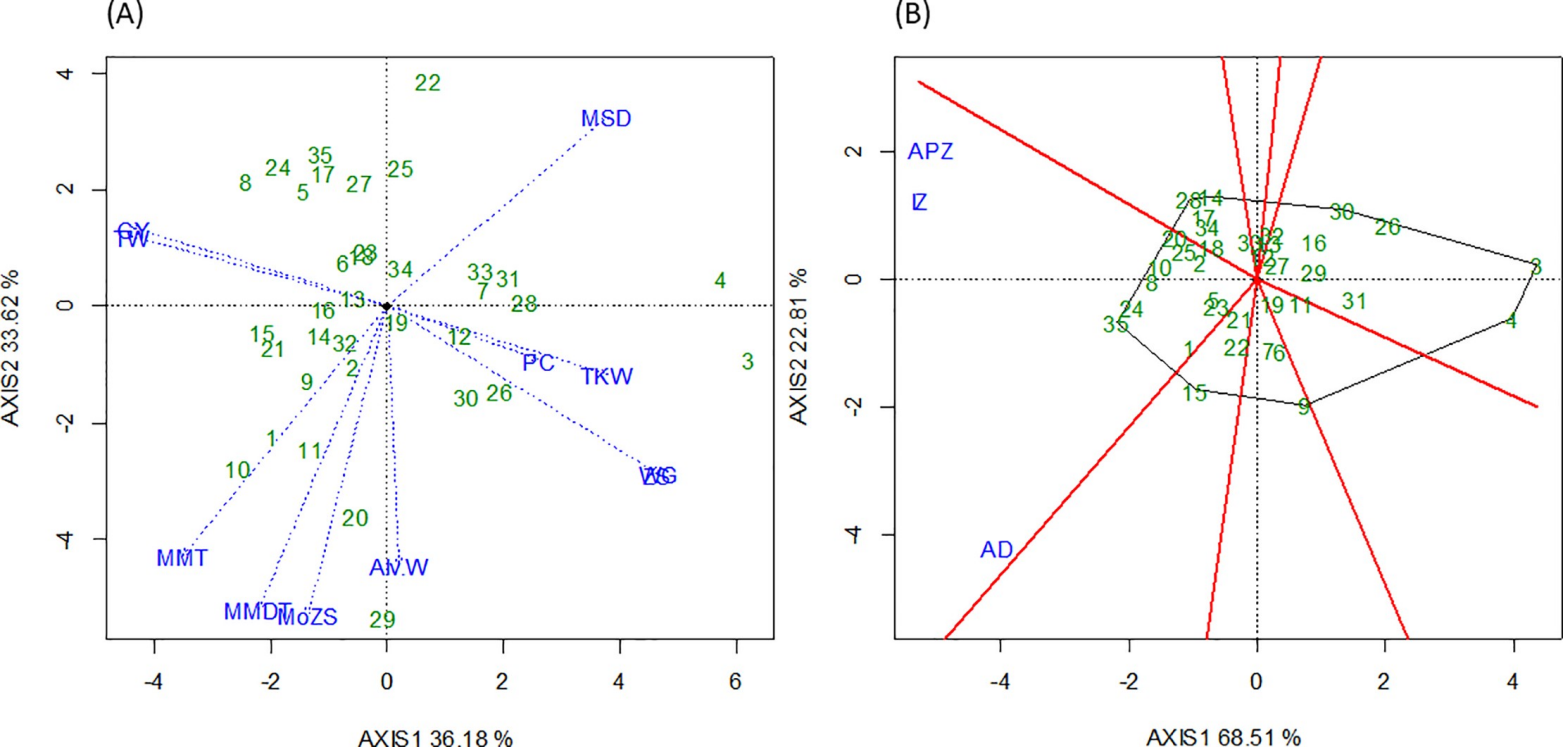
(A) A GGT biplot of the grain yield (GY), thousand kernel weight (TKW), test weight (TW), Zeleny sedimentation value (ZS), modified Zeleny sedimentation value (MZS), protein content (PC), wet gluten content (WGC), alveograph energy value (Alv.W), mixograph developmental time (MDT), mixograph mixing tolerance (MMT), mixograph softening degree (MSD) and (B) GGE biplot for three environment: Adana (AD), Adapazarı (APZ) and Izmir (IZ) for 35 Turkish spring wheat cultivars (Mean of 2009, 2011, and 2012) (refer to Table 1 for the full names of the cultivars).

To see the effect of cultivars and their interaction with traits (G x T) and the environment (G x E) we conducted a GGT/E biplot analysis. The G x T biplot showed that the first two principal components explained 69.8% of the total variation (Fig 1A). The first principal component (PC1 or AXIS 1) explained 36.2% of the variation. The group of traits explaining this variation were alveograph energy value, mixograph developmental time, mixograph mixing tolerance with positive correlation among them and mixograph softening degree with negative effects on the first four components. The second principal component (PC2 or AXIS 2) explained 33.6% of the variation and the group of traits explaining this variation were GY and TW (positive connection), PC, TKW, wet gluten and Zeleny sedimentation values (negative correlation with GY and TW). Traits related to bread making quality are grouped on the first principal axis, showing positive association amongst them and negative association with mixograph softening degree. Overall, PC1 explains the quality traits whereas PC2 explains the yield traits. The cultivars associated with these traits were also grouped according to their performance for respective traits.

To see the performance of individual cultivars for GY in the three environments (locations), we conducted a “Which Won Where/What” GGE biplot analysis (Fig 1B). This biplot divided cultivars according to their suitability to the environment. Cultivars that fall outside of these three environments were not particularly productive. Overall, Adana location was more diverse than Adapazarı and Izmir. The two cultivars which performed best for GY at Adana were Hanli and Adana-99, and at Adapazarı location were Osmaniyem and Gönen-98. At Izmir—Ziyabey-98 and Menemen were the top GY performers. Across locations, the top two yielding cultivars were Ziyabey-98 and Menemen.

### 3.4 Genetic gain for yield components and quality traits

Table 4 shows the mean values, regression coefficient and genetic gain for GY and quality traits for cultivars released during different time periods. Overall, the results show that continuous breeding efforts increased the grain yield and some quality traits in 35 wheat cultivars released between 1964 and 2010. Comparing cultivars released after the year 2000 as a one group to all previous years, there is clear indication that there is yield improvement, suggesting genetic gains over studied breeding period.

**Table 4:** Genetic gain for grain yield (GY) and quality parameters of 35 Turkish spring wheat cultivar released between 1964 and 2010.

Traits	Data years	< 1980	1981–1990	1991–2000	2000+	R^2^	Genetic gain
No. of genotypes		5	9	13	8		
**GY (kg ha**^**-1**^**)**	2009; 2011; 2012	5006	5838	5944	6174	0.41***	30.9 kg yr^-1^
**TKW (g)**	2011,2012	42.8	39.1	40.6	41.0	0.008	
**TW (kg hL**^**-1**^**)**	2009; 2011; 2012	74.1	74.7	74.8	75.0	0.43***	0.24 kg hl^-1^ yr^-1^
**PC (%)**	2009; 2011; 2012	13.6	13.6	13.6	13.5	0.08	
**WGC (%)**	2009; 2011	30.4	27.7	28.1	28.4	0.08	
**ZS (mL)**	2009; 2011; 2012	38.4	35.6	36.0	36.2	0.07	
**Alv.W (10**^**-4**^**joule)**	2009; 2011	176.0	171	208	180	0.05	
**MDT (min)**	2009; 2011; 2012	4.21	5.07	5.78	5.34	0.13*	0.40 min yr^-1^
**MMT (°)**	2009; 2011; 2012	142	147	150	149	0.17*	0.23%yr^-1^
**MSD (MU)**	2009; 2011; 2012	12.4	10.4	10.4	10.5	0.10	

* Significance at 5% (P = 0.05) level, ** 1% (P = 0.01) level

*** 0.1% (P = 0.001) level. Mixograph Unit (MU), grain yield (GY), thousand kernel weight (TKW), test weight (TW), protein content (PC), wet gluten content (WGC), Zeleny sedimentation test value (ZS), alveograph energy value (Alv.W), mixograph developmental time (MDT), mixograph mixing tolerance (MMT), mixograph softening degree (MSD).

**Fig 2:**
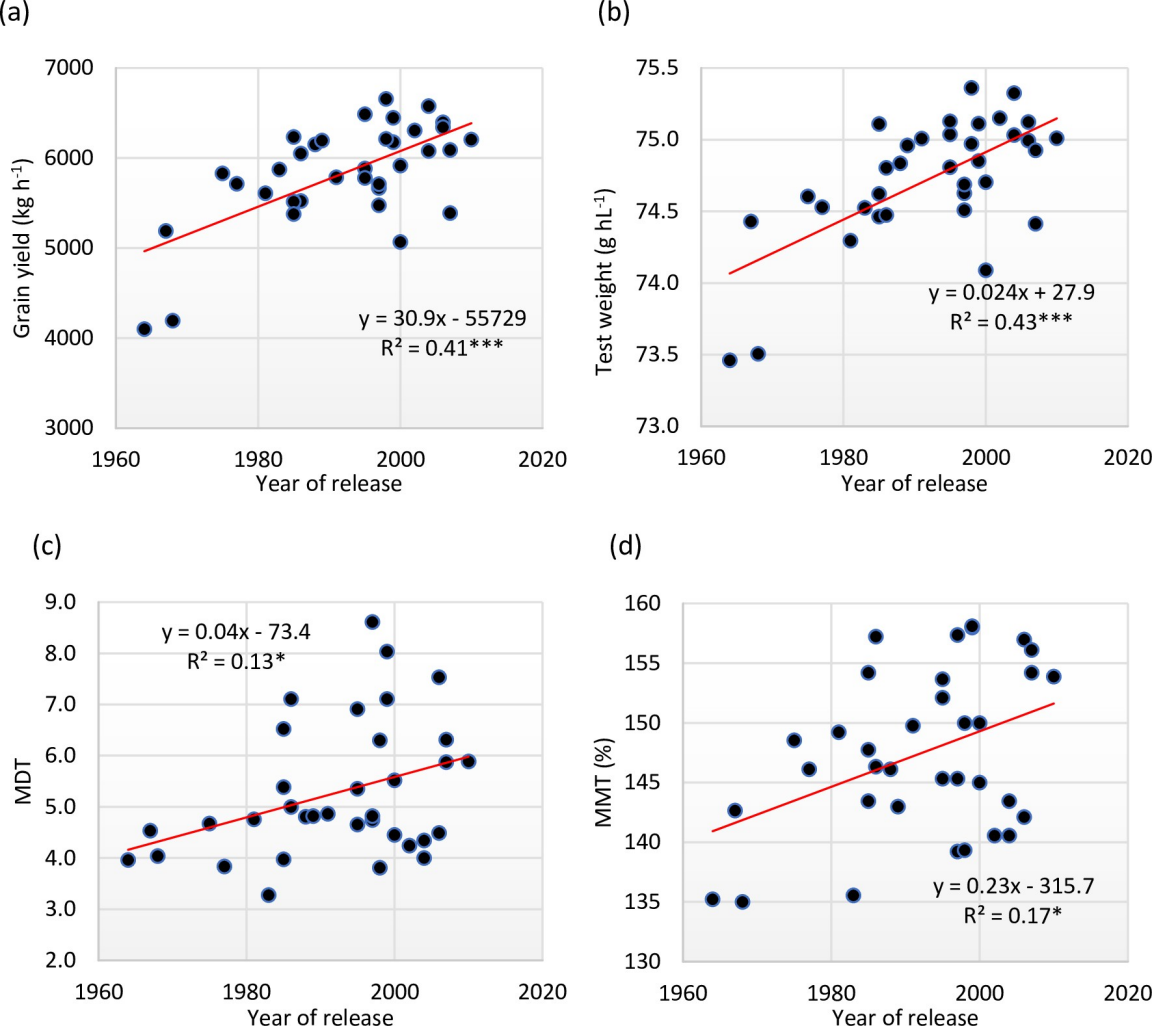
Regression of year of release on (a) grain yield (b) test weight (c) mixograph maximum development time (MDT) (d) mixograph mixing tolerance (MMT) for 35 Turkish spring wheat cultivars released between 1964 and 2010.

Change in important traits from 1964 to 2010 is shown in regression analysis in Fig 2. The regression analysis of GY demonstrates that increases in GY were statistically significant (R^2^ = 0.41, P<0.001) and accounted for 30.9 kg ha^−1^ yr^-1^ (Fig 2A). Therefore, this annual rate of genetic gain was 0.75% in comparison to the yield of Akova B-2 (4102 kg ha^-1^). The other way of determining the genetic gain is comparison of two new cultivars (Hanlı and Aday-1) released after 2006 (6149 kg ha^-1^) with the average yield (6149 kg ha^-1^) of two oldest cultivar (Akova B-2 and Sakarya-75) released in the 1960s. Here, the total annual rate of yield improvement in was 1503 kg ha^-1^ or 33 kg ha^-1^ per year or 0.70% over 46 years of wheat breeding.

For physical traits like TKW and TW, which are responsible for grain milling quality, only TW showed significant improvement over time. The grains become heavier in more recently bred cultivars. (Fig 2B). The results obtained for Zeleny sedimentation value, wet gluten content and alveograph energy value showed no genetic gain over the years. PC showed a slight negative trend, but it was non-significant. Gluten quality and dough development parameters during bread-making process were analyzed by mixograph. The change over the breeding period for mixograph developmental time (0.40 min per year) and mixograph mixing tolerance (0.23.3% yr^-1^) was significant and positive. This significant change demonstrates genetic improvement in the last 46 years for these traits (Fig 2C and 2D).

The highest yielding new variety in this study, Ziyabey-98 (6657 kg ha^-1^), yielded almost twice of the older variety, Akova B-2 (4102 kg ha^-1^). Ziyabey-98 showed the highest test weight whereas Akova B-2 had the lowest. Osmaniyem (released in 2006) showed the highest TKW and Alta-81 (released in 1981) showed the lowest TKW. Overall, the grain quality parameters slightly deteriorated with increasing GY. Two oldest cultivars, Akova B-2 and Aköz-867, showed low yield but had above average protein content (13.6% and 13.7% respectively) and better values than other cultivars for quality traits such as Zeleny sedimentation (37.6 ml and 33.4 ml respectively) and wet gluten content (30.1% and 29.47% respectively). Two new cultivars, Osmaniyem (13.75%) and Beşköprü (13.32%), had lower protein content than the two old cultivars (Akova B-2 and Aköz-867), an average alveograph energy value, shorter mixograph development time and lower mixing tolerance. (S2 Table).

### 3.5 Composition of HMW-GS and LMW-GS and its effect on quality parameters

The numbers of loci and alleles and the frequency of HMW-GSs observed in 35 cultivars are shown in Table 5. At *Glu-A1* locus, two alleles were recorded and most common one was subunit 2* (allele b) with a frequency of 67%. Next highest frequency was reported for subunit 1 (allele a) and it was 33%. At *Glu-B1* three alleles were detected: 17+18 (allele i), 7 + 9 (allele c) and 7+8 (allele b) with respective frequency of 30%, 45% and 18%. At locus *Glu-D1*, subunit combinations 2+12 (allele a) and 5+10 (allele d) were observed with respective frequency of 33% and 67%.

**Table 5:** Allelic frequency and the effect of HMW-GS and LMW-GS on grain yield and quality parameters studied on 35 Turkish spring wheat cultivars (mean across years for each subunit).

Glu Locus	Subunit	No.	F (%)	GY (kg ha^-1^)	TKW (g)	TW (kg hl^-1^)	PC (%)	WGC (%)	ZS (ml)	Alv.W (Joule)	MDT (min)	MMT (°)	MSD (MU)
*Glu-A1*	1	11	33	5985	40.8	74.8	13.6	28.1	36.0	190.1	5.6	149.2	10.6
	2*	22	67	5738	40.8	74.7	13.6	28.6	36.5	188.1	5.1	146.3	10.8
*Glu-B1*	17+18	10	30	5932	40.3	74.8	13.6	28.3	36.1	209.4	6.4	152.8	9.6
	7+8	6	18	5319	40.6	74.3	13.6	29.8	38.0	182.2	5.3	147.5	10.8
	7+9	15	45	5875	41.1	74.7	13.6	28.1	36.0	181.8	4.8	144.6	11.1
*Glu-D1*	2+12	11	33	5440	42.1	74.5	13.6	29.1	37.0	198.9	5.0	144.5	11.8
	5+10	22	67	6022	40.1	74.8	13.6	28.1	36.0	183.8	5.5	148.7	10.2
*Glu-A3*	b	7	21	5859	40.0	74.8	13.6	28.7	36.6	200.9	5.6	149.2	9.9
	c	19	58	5737	41.0	74.7	13.6	28.2	36.0	190.2	5.3	146.5	11.0
	f	4	12	6031	41.1	74.8	13.5	28.4	36.2	163.1	5.1	148.8	9.8
*Glu-B3*	b	6	18	5770	41.2	74.7	13.6	27.9	35.8	179.9	5.0	149.6	9.9
	g	5	15	5821	40.8	74.7	13.5	29.3	37.1	208.4	6.7	152.4	10.3
	h	7	21	6015	40.0	74.9	13.5	28.7	36.6	189.6	5.2	145.5	11.4
	i	12	36	5889	41.1	74.8	13.6	28.0	35.8	185.9	4.9	145.3	11.0
*Glu-D3*	a	6	18	5266	40.4	74.3	13.6	30.0	38.2	196.5	5.7	147.7	10.3
	b	21	64	6063	40.0	74.9	13.5	27.7	35.5	178.4	5.1	147.3	10.7
	c	6	18	5570	43.6	74.6	13.6	29.4	37.3	217.6	5.6	147.0	11.3
1B/1R	-	28	85	5782	40.8	74.7	13.6	28.4	36.3	190.0	5.4	147.6	10.8
	+	5	15	6087	40.8	74.7	13.6	28.4	36.3	188.8	5.3	147.3	10.7

Grain yield (GY), thousand kernel weight (TKW), test weight (TW), protein content (PC), wet gluten content (WGC), Zeleny sedimentation value (ZS), alveograph energy value W (Alv.W), mixograph developmental time (MDT), mixograph mixing tolerance (MMT), mixograph softening degree (MSD), Frequency (F). Varieties Orso and Aday-1 were not included into analysis.

A total score (TS) [38] was given for each subunit to quantify the effect of HMW-GSs on quality parameters (S3 Table). The cultivars score varied between 6 and 10. The scores 9 and 10 were most common as the higher frequency was reported for 2* allele in *Glu-A1*, 7 + 9 alleles in *Glu-B1* and 5 + 10 alleles in *Glu-D1*. There was positive association of TS with the mixograph developmental time (R^2^ = 0.20, P = 0.008) and mixograph mixing tolerance (R^2^ = 0.27, P = 0.002), and a negative association with mixograph softening degree (R^2^ = -0.29, P = 0.001).

Loci number and allele frequency for LMW-GSs in 20 cultivars is presented in Table 5. At *Glu-A3*, three alleles were reported with the frequency of allele c 58%. At *Glu-B3*, four alleles were reported with allele i being most frequent (37%) followed by allele h (21%), allele b (18%) and allele g (15%). At *Glu-D3*, three alleles were reported and highest frequency was for allele b (64%) and followed by alleles a and c with similar frequency (18%).

The current understanding of the effects of low molecular weight glutenin subunits on the grain quality is low as compared to the high molecular weight glutenin subunits. In this study Alleles *Glu-A3b*, *Glu-B3d*, *Glu-B3h* were reported to be present in the old as well as in modern cultivars and did not show any specific tendency of increase or decrease over time. Only five cultivars, one each from groups 3 and 4 and three from the modern group possessed 1B.1R translocation (Table 5).

A change in frequency of HMW-GS, LMW-GS and 1B/1R translocation over the breeding period in 35 spring wheat cultivars in Turkey is shown in S4 Table. The frequency of *Glu-A1* subunit 1 was 0% in the first breeding period, around 32% in second and third (33.3% and 30.8% respectively), and increased in the modern group up to 57.1%, showing clear improvement for these loci. In contrast, subunit 2* showed a continued decrease over all the periods. A similar trend of decrease in frequency was also seen for *Glu-B1* loci for subunit 7+8 for first three breeding period, completely disappearing in the third group and slightly increasing again in the modern group. The *Glu-D1* loci subunits 2+12 and 5+10 showed an overall decrease and increase over the 46 years of breeding period, respectively. The frequency of 1B/1R translocation was higher in the modern group of cultivars.

## 4. Discussion

### 4.1 Genotype x environment interaction (GEI)

In this study, grain yield and quality traits were strongly influenced by the genetics of the cultivars and by the environment (primarily the testing locations). As the set of cultivars used in this experiment was the same in all three locations, any difference in response of cultivars for various traits was, therefore, mainly due to G x E interaction. However, only test weight seems to have been significantly influenced by the interaction of G x E. Grain yield was more affected by cultivar than any other factor. The significant change in the performance of 35 cultivars for GY in different locations suggests that the breeding programs were able to successfully develop adoptive cultivars for specific locations. However, the “Which Won Where/What” biplot analysis shows that less than 50% of cultivars fall in the group of suitable for either of these three regions, with the rest of the cultivars not being ideal for any location. This may be the reason for a lack of strong cultivars by testing location interaction for most of the traits.

All three testing locations are in the coastal areas of Turkey and have some similarity in weather patterns. These regions are suitable for spring wheat production in Turkey. However, they have different sowing dates, soil conditions and rainfall, which seem to influence the interaction. This strong effect of testing location is expressed on all the traits studied. Due of this strong regional effect, some cultivars suitable for one location may not to be suitable for other locations. This could also be related to significant differences among breeding programs and breeding program x variety interactions for all traits studied in this experiment, except for protein content. Protein content was mostly influenced by the testing location, showing the trait’s sensitivity to the environment, as has been reported previously [39] and [40].

Biplot analysis clearly shows that Adana is the best location for grain yield, possibly due to higher rainfall. Izmir showed relatively higher TKW, which is negatively associated with GY, possibly explaining lower yield in Izmir than Adana. The nature of negative association between GY and TKW in this experiment is not very clear but one possible explanation could be that low rainfall in Izmir limited the grain number, which may have increased the weight of a single grain. Turkey generally experiences moisture and heat stress during the grain-filling period, which may affect fruiting efficiency. Such a trend of negative association has also been reported [26] in Turkish winter bread wheat cultivars in two locations (Konya and Eskişehir) out of five.

Quality traits like alveograph energy value-W (indicating gluten strength and extension properties), wet gluten (indicating protein content) and Zeleny sedimentation value (indicating protein quality)—were relatively better in Adapazari than in Izmir and Adana. These traits are strongly influenced by environmental conditions, largely by the availability and uptake of nitrogen. Adapazarı may have better soil conditions, with higher total available nitrogen. As we reported for spring wheat [25], if nitrogen-utilization efficiency (grain dry matter yield per unit of crop nitrogen uptake at harvest) is constant, then any higher nitrogen or protein content in the grain is related to higher nitrogen uptake by the plant. Adapazarı also benefits from a relatively longer growing period, longer grain filling period, probably deeper root system, and higher nitrogen take-up, resulting in better grain quality.

Whereas cultivars from Adapazarı showed higher grain quality and poorer yield, cultivars from Adana showed higher grain yield, possibly at the cost of quality. Biplot analysis shows a trade-off between yield (GY and TW) and protein related quality traits (protein content, wet gluten and Zeleny sedimentation) and lack of strong association between yield and other flour and dough quality traits (alveograph energy value, mixograph developmental time, mixograph softening degree and mixograph mixing tolerance). Negative correlation between GY and Zeleny sedimentation value and between GY and PC in Turkish wheat cultivars has been reported previously [40]. Generally, negative association between GY and PC is a well-documented fact and reported by many authors worldwide [19,41–43]. This is a challenge for the development of cultivars eligible for superior quality grades and high yield.

At the variety level, such associations are explained by the fact that old cultivars like Akova B-2 and Aköz-867, developed by the Adapazarı breeding program, were low-yielding but better in protein quality (Zeleny sedimentation, wet gluten). High yielding cultivars like Ziyabey-98 and Menemen, developed by Izmir breeding program, were poorer in quality traits (Zeleny sedimentation, wet gluten and maximum mixograph developmental time). Overall biplot analysis was very effective in studying G x E interaction. GGE biplot analysis gave a clear grouping of traits according to yield and quality. It also helped identify specific cultivars for particular traits. These results are in agreement with other studies [40, 44] indicating that the grouping of traits is associated with genotype performance. Three cultivars which performed better for grain yield across locations (Ziyabey-98, Menemen and Basribey-95) could be recommended for stable spring wheat production across the studied regions because of their wider adaptability; whereas Pamukova-97 could be recommended for future breeding to improve grain quality parameters.

### 4.2 Breeding progress over 46-year period

Apart from studying G x E interaction, other objective of this study was to analyze improvement in yield and quality traits during 46 years of wheat breeding at three breeding programs (Adana, Adapazarı and Izmir). Historically, improvement in grain yield may have compromised traits such as grain quality, especially related to the trade-off between yield and protein content [43,45,46]. Overall, the progress in grain yield improvement was very substantial: from around 4 t ha^-1^ before the 1960s to around 6 t ha^-1^ after 2000. This improvement was achieved with approximately 30.9 kg h^-1^ yr^-1^ (0.70% yr^-1^; R^2^ = 0.41, P < 0.001). This yield increase is comparable to similar studies in other regions [23,25,47]. For example, studies [23,47] have reported an average grain yield increase of 0.7 to 0.95% during the past 100 years in spring bread wheat. Another study [48] reported a continuous increase of genetic gain for grain yield of 30 kg ha^−1^ yr^−1^ (0.59% yr^−1^; R2 = 0.58, P = 0.01) in spring wheat cultivars developed by CIMMYT from 1966 to 2009. Research [49] also shows the increase of 0.45% per year during 28 years of wheat breeding (from 1978 to 2006) in Mediterranean region.

Despite continued genetic gain in GY for the whole period, this study demonstrated that there was no significant change in the rate of genetic gain among cultivars released after 1990, results that will need to be addressed in the breeding programs. Historically, until the 1980s, wheat breeding programs in Turkey mainly focused on yield and yield components. After 1980, breeders recognized the importance wheat quality, and since then wheat breading programs have focused on grain quality along with grain yield. Disease resistance breeding also increased in importance. In this study, quality parameters showed improvement after 1980s. Several varieties released in Turkey originated from CIMMYT. Annually CIMMYT provides international nurseries for selection of superior lines and potential release as varieties. However, the success of this work to large extend depends on the vigorous and robust breeding system able to screen hundreds of entries to identify the best. Spring wheat varieties originated from CIMMYT germplasm, selected and released by Turkish breeders represents the cooperative product for the benefit of farmers and consumers.

As the industrial process of bread making is fully mechanized and automated, strong gluten, characterized by higher alveograph energy value, higher development time and mixing tolerance, is required. For example, if flour takes long time to mix, it is considered undesirable as it consumes electric power and is not economical. Also, good mixing tolerance of dough is important for a baker as it gives broader “window of opportunity” to stop the process of mixing at optimum stage of dough development. Elasticity of dough is another important prerequisite for good bread making. In this experiment, significant increase over the period of breeding was observed for mixograph developmental time and mixing tolerance, which increased constantly until 2000 and then remain unchanged during the modern period. A similar pattern of improvement in wheat dough quality traits was reported by Guzmán [24]. Similar rate of genetic gain was also reported in other studies for similar traits [23,42]. This change partly can be explain by the change in protein quality and glutenins composition [19,23].

As this set of cultivars did not show any increase in protein content over time, the improvement in mixing time could be related to an improvement in protein quality. Our data showed an increase in frequency of HMW-GS subunits 5+10, which generally have positive impact on bread-making quality [38]. Previous studies reported that HMW-GSs have the highest effect on the dough and bread-making rheological properties [23,50–52]. It was also reported that the most important role is played by alleles 1 and 2* of *Glu-A1* in comparison to the null allele [15,53,54]. Dough strength depends on 5+10 alleles of the *Glu-D1* [55]. In this set of cultivars, allele 5+10 frequency increased and 2+12 decreased over 46 years of Turkish wheat breeding.

## 5. Conclusions

The present study showed that grain and quality traits were mainly influenced by G (cultivars) and E (testing location), with the effect of G being more important. Therefore, breeding for higher GY and dough quality should focus on developing cultivars with strong genetic potential. There was a trade-off between GY and PC but there was also enhancement in grain quality over years, due to a change in protein composition. Future breeding efforts to improve bread making quality may focus on improvement in HMW-GS rather than protein content. Also, in future investigations the role of soil nutrient, nitrogen uptake and root system on grain quality and protein composition could give valuable information, as there may be an association between nitrogen use efficiency and protein composition.

## Supporting information

S1 Fig Genotype main effect plus genotype x environment effect (GGE) biplot for GY in for 35 Turkish spring wheat cultivars (Mean of 2009, 2011, and 2012) (refer to Table 1 for the full names of the cultivars). (DOCX)

S1 Table ANOVA results showing per cent variance and significance for yield and quality traits for 35 Turkish spring wheat cultivars studded in 2009, 2011 and 2012. (DOCX)

S2 Table Performance of 35 Turkish spring wheat cultivars released between 1964 still 2010 (means across years and locations). (DOCX)

S3 Table List of alleles found in 35 cultivars and their total score for HMW-GS and 1B/1R translocation. (DOCX)

S4 Table Frequency of HMW-GS and LMW-GS and the 1B/1R translocation in 35 spring wheat cultivars release between 1964 to 2010 in Turkey. (DOCX)

S1 Dataset Wheat grain quality data CIMMYT Turkey. (XLSX)

S2 Dataset Wheat yield data CIMMYT Turkey. (XLSX)
