# Supplementary material for: Genotype x environment interaction and genetic gain for grain yield and grain quality traits in Turkish spring wheat released between 1964 and 2010
Source: PLoS One. 2019 Jul 18;14(7):e0219432. doi: 10.1371/journal.pone.0219432 (PMC6638857; doi:10.1371/journal.pone.0219432)
Supplement: S1 Fig — (DOCX) [file pone.0219432.s001.docx]

**Supplementary Figure 1. Genotype main effect plus genotype x environment effect (GGE) biplot for GY in for 35 Turkish spring wheat cultivars (Mean of 2009, 2011, and 2012) (refer to Table 1 for the full names of the cultivars).**
