# Supplementary material for: Genotype x environment interaction and genetic gain for grain yield and grain quality traits in Turkish spring wheat released between 1964 and 2010
Source: PLoS One. 2019 Jul 18;14(7):e0219432. doi: 10.1371/journal.pone.0219432 (PMC6638857; doi:10.1371/journal.pone.0219432)
Supplement: S1 Table — (DOCX) [file pone.0219432.s002.docx]

**Supplementary Table 1. ANOVA results showing per cent variance and significance for yield and quality traits for 35 Turkish spring wheat cultivars studded in 2009, 2011 and 2012**.

| **Variables** | **GY** | **TKW** | **TW** | **PC** | **WGC** | **ZS** | **MZS** | **Alv.W** | **MDT** | **MMT** | **MSD** |
| --- | --- | --- | --- | --- | --- | --- | --- | --- | --- | --- | --- |
| TL | 16.7 *** | 23.8 *** | 97.6 *** | 77.1 *** | 25.7 *** | 24.8 *** | 4.55 *** | 16.6 *** | 3.50 *** | 8.09 *** | 9.56 *** |
| BP | 8.68 *** | 5.40 *** | 0.20 *** | 0.10 NS | 4.19 *** | 4.33 *** | 14.9 *** | 5.27 *** | 3.30 *** | 5.63 *** | 1.06 * |
| TL x BP | 2.88 *** | 0.43 NS | 0.25 *** | 1.36 *** | 0.89 NS | 1.45 ** | 0.87 NS | 0.11 NS | 0.48 NS | 0.70 NS | 3.01 ** |
| BP x Cul | 13.3 *** | 26.5 *** | 0.35 *** | 1.45 NS | 15.4 *** | 14.7 *** | 34.8 *** | 33.0 *** | 19.4 *** | 31.4 *** | 18.8 *** |
| TLx BPx Cul | 7.91 NS | 2.27 NS | 0.49 *** | 4.30 NS | 5.77 NS | 6.47 NS | 5.55 NS | 11.4 NS | 4.35 NS | 6.28 NS | 10.6 NS |
| **Covariates** |  |  |  |  |  |  |  |  |  |  |  |
| Year | 23.2 *** | 13.9 *** | 0.36 *** | 1.67 *** | 21.2 *** | 22.1 *** | 7.14 *** | 0.02 NS | 39.8 *** | 9.34 *** | 15.7 *** |
| **Residual** | 27.4 | 27.62 | 0.74 | 14.07 | 26.92 | 26.11 | 40.08 | 33.6 | 30.35 | 39 | 41.71 |

TL= Testing location; BP= Breeding program; Cul= Cultivar; ***= <0.001; **= <0.05; *= <0.5 level of significance.
