# Supplementary material for: Genotype x environment interaction and genetic gain for grain yield and grain quality traits in Turkish spring wheat released between 1964 and 2010
Source: PLoS One. 2019 Jul 18;14(7):e0219432. doi: 10.1371/journal.pone.0219432 (PMC6638857; doi:10.1371/journal.pone.0219432)
Supplement: S2 Table — (DOCX) [file pone.0219432.s003.docx]

**Supplementary Table 2. Performance of 35 Turkish spring wheat cultivars released between 1964 still 2010 (means across years and locations).**

| Cultivars | OC | YoR | GY (kg ha^-1^) | TKW (g) | TW (kg L^-1^) | PC% | WGC (%) | ZS (ml) | MZS (ml) | Alv.W (Joule) | MDT (min) | MMT (°) | MSD (%) |
| --- | --- | --- | --- | --- | --- | --- | --- | --- | --- | --- | --- | --- | --- |
| Adana 99 | ADA | 1999 | 6177 | 39.25 | 74.85 | 13.43 | 28.38 | 35.92 | 61.67 | 215.5 | 7.11 | 158.0 | 9.00 |
| Aday-1 | ADA | 2010 | 6207 | 41.88 | 75.01 | 13.59 | 29.13 | 37.07 | 52.33 | 187.8 | 5.89 | 153.8 | 10.00 |
| Akova B-2 | ADPZ | 1964 | 4102 | 44.14 | 73.46 | 13.72 | 33.90 | 42.50 | 44.83 | 197.6 | 3.97 | 135.2 | 12.89 |
| Aköz-867 | ADPZ | 1968 | 4194 | 44.52 | 73.51 | 13.60 | 32.62 | 40.85 | 36.50 | 175.0 | 4.04 | 135.0 | 14.88 |
| Alibey | IZM | 2004 | 6078 | 36.84 | 75.03 | 13.48 | 26.55 | 34.11 | 34.83 | 177.6 | 4.34 | 143.4 | 10.00 |
| Ata81 | IZM | 1981 | 5612 | 35.94 | 74.30 | 13.50 | 27.16 | 35.00 | 41.67 | 168.4 | 4.76 | 149.2 | 9.44 |
| Bandırma-97 | ADPZ | 1997 | 5667 | 44.82 | 74.69 | 13.41 | 29.38 | 37.31 | 45.17 | 234.3 | 4.82 | 139.2 | 14.11 |
| Basribey95 | IZM | 1995 | 6487 | 36.77 | 75.13 | 13.54 | 25.26 | 32.69 | 37.17 | 161.2 | 4.66 | 145.3 | 9.44 |
| Beşköprü | ADPZ | 2007 | 5391 | 41.85 | 74.41 | 13.32 | 27.12 | 34.76 | 54.83 | 184.4 | 6.31 | 156.1 | 8.00 |
| Ceyhan 99 | ADA | 1999 | 6448 | 41.96 | 75.11 | 13.58 | 27.26 | 34.97 | 64.67 | 221.0 | 8.03 | 158.1 | 7.78 |
| Çukurova-86 | ADA | 1986 | 5522 | 39.36 | 74.48 | 13.54 | 28.03 | 35.84 | 58.67 | 206.0 | 7.11 | 157.2 | 7.11 |
| Cumhuriyet 75 | IZM | 1975 | 5829 | 45.63 | 74.60 | 13.65 | 29.74 | 37.52 | 46.67 | 188.8 | 4.68 | 148.5 | 10.33 |
| Doğankent 1 | ADA | 1991 | 5789 | 38.42 | 75.01 | 13.58 | 28.08 | 35.97 | 52.50 | 177.2 | 4.87 | 149.7 | 11.33 |
| Gönen98 | IZM | 1998 | 6214 | 39.42 | 74.97 | 13.74 | 27.14 | 35.05 | 50.67 | 192.3 | 6.30 | 150.0 | 10.00 |
| Hanlı | ADPZ | 2007 | 6091 | 40.53 | 74.93 | 13.31 | 27.29 | 35.30 | 54.67 | 158.4 | 5.88 | 154.2 | 8.22 |
| İrnerio | ADPZ | 1985 | 5515 | 37.45 | 74.62 | 13.65 | 26.78 | 34.66 | 48.33 | 161.6 | 5.39 | 154.2 | 9.22 |
| İzmir81 | IZM | 1985 | 6235 | 37.32 | 75.11 | 13.66 | 26.62 | 34.23 | 34.50 | 158.7 | 3.98 | 143.4 | 10.33 |
| Kaklıc88 | IZM | 1988 | 6152 | 38.35 | 74.84 | 13.66 | 28.42 | 36.28 | 42.83 | 142.3 | 4.81 | 146.1 | 9.33 |
| Karacabey-97 | ADPZ | 1997 | 5710 | 42.37 | 74.63 | 13.53 | 27.65 | 35.47 | 40.83 | 265.4 | 4.74 | 145.3 | 10.22 |
| Karatopak | ADA | 2006 | 6396 | 40.75 | 75.12 | 13.64 | 31.29 | 39.48 | 68.00 | 232.0 | 7.53 | 157.0 | 9.89 |
| Kaşifbey95 | IZM | 1995 | 5886 | 37.48 | 74.81 | 13.47 | 26.88 | 34.49 | 50.33 | 198.2 | 6.91 | 153.6 | 9.89 |
| Libellula | ADPZ | 1983 | 5876 | 37.51 | 74.52 | 13.48 | 27.07 | 34.96 | 27.00 | 138.8 | 3.28 | 135.5 | 14.44 |
| Maramara86 | IZM | 1986 | 6051 | 39.70 | 74.81 | 13.52 | 28.02 | 35.88 | 42.83 | 162.8 | 5.00 | 146.3 | 11.22 |
| Menemen | IZM | 2004 | 6577 | 37.51 | 75.32 | 13.45 | 26.36 | 33.95 | 37.00 | 179.4 | 4.00 | 140.5 | 10.44 |
| Meta2002 | IZM | 2002 | 6309 | 41.18 | 75.15 | 13.59 | 27.61 | 35.26 | 39.83 | 162.6 | 4.24 | 140.5 | 15.11 |
| Momtchill | ADPZ | 2000 | 5070 | 45.03 | 74.09 | 13.76 | 29.22 | 37.42 | 51.00 | 197.3 | 5.52 | 150.0 | 10.11 |
| Orso | ADA | 1977 | 5715 | 37.58 | 74.53 | 13.64 | 26.37 | 33.87 | 36.17 | 144.1 | 3.84 | 146.1 | 10.50 |
| Osmaniyem | ADA | 2006 | 6342 | 47.12 | 74.99 | 13.75 | 31.65 | 39.94 | 48.00 | 159.2 | 4.49 | 142.1 | 12.56 |
| Pamukova-97 | ADPZ | 1997 | 5477 | 36.41 | 74.51 | 13.60 | 32.26 | 40.68 | 63.67 | 293.9 | 8.61 | 157.3 | 6.88 |
| Pandas | ADA | 1985 | 5381 | 44.46 | 74.46 | 13.74 | 29.62 | 37.34 | 49.83 | 202.4 | 6.53 | 147.7 | 10.13 |
| Sakarya-75 | ADPZ | 1967 | 5191 | 44.40 | 74.43 | 13.53 | 29.54 | 37.04 | 47.50 | 174.3 | 4.53 | 142.6 | 13.56 |
| Seyhan 95 | ADA | 1995 | 5779 | 42.60 | 75.04 | 13.59 | 27.34 | 35.13 | 54.50 | 207.2 | 5.36 | 152.1 | 10.89 |
| Tahirova-2000 | ADPZ | 2000 | 5917 | 42.47 | 74.70 | 13.67 | 29.76 | 38.01 | 40.33 | 188.4 | 4.46 | 145.0 | 13.33 |
| Yüreğir-89 | ADA | 1989 | 6196 | 41.98 | 74.96 | 13.68 | 27.90 | 35.77 | 48.67 | 193.3 | 4.82 | 143.0 | 12.67 |
| Ziyabey98 | IZM | 1998 | 6656 | 41.38 | 75.36 | 13.41 | 26.95 | 34.59 | 40.50 | 154.2 | 3.81 | 139.3 | 11.78 |
| Minimum |  |  | 4102 | 35.94 | 73.46 | 13.31 | 25.26 | 32.69 | 27.00 | 138.8 | 3.28 | 135.0 | 6.88 |
| Maximum |  |  | 6656 | 47.12 | 75.36 | 13.76 | 33.90 | 42.50 | 68.00 | 293.97 | 8.61 | 158.1 | 15.11 |
| Average |  |  | 5835 | 40.70 | 74.73 | 13.57 | 28.41 | 36.27 | 47.10 | 187.52 | 5.27 | 147.4 | 10.72 |
