# Supplementary material for: Genotype x environment interaction and genetic gain for grain yield and grain quality traits in Turkish spring wheat released between 1964 and 2010
Source: PLoS One. 2019 Jul 18;14(7):e0219432. doi: 10.1371/journal.pone.0219432 (PMC6638857; doi:10.1371/journal.pone.0219432)
Supplement: S3 Table — (DOCX) [file pone.0219432.s004.docx]

**Supplementary Table 3. List of alleles found in 35 cultivars and their total score for HMW-GS and 1B/1R translocation.**

|  | HMW Glutenins | | |  | LMW Glutenins | | |  |
| --- | --- | --- | --- | --- | --- | --- | --- | --- |
| C/M Pedigri | Glu- A1 | Glu-B1 | Glu-D1 | Score for HMW | Glu-A3 | Glu-B3 | Glu-D3 | Status 1B/1R |
| Adana 99 | 1 | 17+18 | 5+10 | 10 | c | g | b | 1B/1B |
| Akova B-2 | 2* | 7+8 | 2+12 | 8 | c | e | a | 1B/1B |
| Aköz-867 | 2* | 7+8 | 2+12 | 8 | c | g | a | 1B/1B |
| Alibey | 2* | 7+9 | 5+10 | 9 | b | j | b | 1B/1R |
| Ata81 | 2* | 7+8 | 2+12 | 8 | b | b | b | 1B/1B |
| Bandırma-97 | 2* | 7+9 | 2+12 | 7 | c | h | c | 1B/1B |
| Basribey95 | 1 | 7+9 | 5+10 | 9 | c | i | b | 1B/1B |
| Beşköprü | 1 | 7+9 | 5+10 | 9 | c | i | b | 1B/1B |
| Ceyhan 99 | 1 | 17+18 | 5+10 | 10 | c | g | b | 1B/1B |
| Çukurova-86 | 2* | 17+18 | 5+10 | 10 | b | j | b | 1B/1R |
| Cumhuriyet 75 | 2* | 17+18 | 5+10 | 10 | f | b | b | 1B/1B |
| Doğankent 1 | 0 | 17+18 | 2+12 | 6 | c | h | b | 1B/1B |
| Gönen98 | 2* | 17+18 | 2+12 | 8 | c | f | a | 1B/1B |
| Hanlı | 2* | 7+8 | 5+10 | 10 | f | b | a | 1B/1B |
| İrnerio | 1 | 7+8 | 5+10 | 10 | c | e | a | 1B/1B |
| İzmir81 | 2* | 7+9 | 5+10 | 9 | c | b | b | 1B/1B |
| Kaklıc88 | 2* | 7+9 | 5+10 | 9 | f | h | b | 1B/1B |
| Karacabey-97 | 1 | 7+9 | 2+12 | 7 | c | i | c | 1B/1B |
| Karatopak | 1 | 7+8 | 5+10 | 10 | d | g | c | 1B/1B |
| Kaşifbey95 | 2* | 17+18 | 5+10 | 10 | c | g | b | 1B/1B |
| Libellula | 1 | 7+9 | 5+10 | 9 | e | i | b | 1B/1B |
| Maramara86 | 2* | 7+9 | 5+10 | 9 | f | h | b | 1B/1B |
| Menemen | 2* | 7+9 | 5+10 | 9 | c | j | b | 1B/1R |
| Meta2002 | 1 | 7 | 5+10 | 8 | c | h | b | 1B/1B |
| Momtchill | 2* | 7+9 | 5+10 | 9 | c | b | c | 1B/1B |
| Osmaniyem | 1 | 7+9 | 5+10 | 9 | b | j | b | 1B/1R |
| Pamukova-97 | 2* | 17+18 | 5+10 | 10 | b | h | a | 1B/1B |
| Pandas | 1 | 7+9 | 2+12 | 7 | c | i | c | 1B/1B |
| Sakarya-75 | 2* | 7+9 | 2+12 | 7 | c | i | c | 1B/1B |
| Seyhan 95 | 2* | 17+18 | 2+12 | 8 | b | b | b | 1B/1B |
| Tahirova-2000 | 2* | 7+9 | 5+10 | 9 | e | j | b | 1B/1R |
| Yüreğir-89 | 2* | 17+18 | 2+12 | 8 | b | i | b | 1B/1B |
| Ziyabey98 | 2* | 7 | 5+10 | 8 | c | h | b | 1B/1B |
