# Supplementary material for: Genotype x environment interaction and genetic gain for grain yield and grain quality traits in Turkish spring wheat released between 1964 and 2010
Source: PLoS One. 2019 Jul 18;14(7):e0219432. doi: 10.1371/journal.pone.0219432 (PMC6638857; doi:10.1371/journal.pone.0219432)
Supplement: S4 Table — (DOCX) [file pone.0219432.s005.docx]

**Supplementary Table 4. Frequency of HMW-GS and LMW-GS and the 1B/1R translocation in 35 spring wheat cultivars release between 1964 to 2010 in Turkey.**

| Glu locus | Subunit | <1980 | 1981-1990 | 1991-2000 | 2000+ |
| --- | --- | --- | --- | --- | --- |
| Glu-A1 | **1** | 0 | 33.3 | 30.8 | 57.1 |
|  | **2*** | 100.0 | 66.7 | 61.5 | 42.9 |
| Glu-B1 | **7+8** | 50.0 | 22.2 | 0.0 | 14.3 |
|  | **7+9** | 25.0 | 55.6 | 23.1 | 57.1 |
|  | **17+18** | 25.0 | 22.2 | 38.5 | 0 |
| Glu-D1 | **2+12** | 75.0 | 33.3 | 38.5 | 0 |
|  | **5+10** | 25.0 | 66.7 | 61.5 | 100.0 |
| Glu-A3 | **b** | 0 | 33.3 | 15.4 | 28.6 |
|  | **c** | 75.0 | 22.2 | 76.9 | 42.9 |
|  | **e** | 0 | 11.1 | 7.7 | 0.0 |
|  | **f** | 25.0 | 22.2 | 0.0 | 14.3 |
| Glu-B3 | **b** | 25.0 | 22.2 | 15.4 | 14.3 |
|  | **e** | 25.0 | 11.1 | 0 | 0 |
|  | **f** | 0 | 0 | 7.7 | 0 |
|  | **g** | 25.0 | 0 | 23.1 | 14.3 |
|  | **h** | 0 | 22.2 | 30.8 | 14.3 |
|  | **i** | 25.0 | 44.4 | 23.1 | 57.1 |
| Glu-D3 | **a** | 50.0 | 11.1 | 15.4 | 14.3 |
|  | **b** | 25.0 | 77.8 | 61.5 | 71.4 |
|  | **c** | 25.0 | 11.1 | 23.1 | 14.3 |
| 1B/1R | **+** | 0 | 11.1 | 7.7 | 42.9 |
